# ADVANCING GLOBAL HEALTHY AGING: MY JOURNEY IN GERONTOLOGICAL RESEARCH

**DOI:** 10.1093/geroni/igae098.0669

**Published:** 2024-12-31

**Authors:** Bei Wu

**Affiliations:** New York University, New York, New York, United States

## Abstract

In this presentation, Dr. Bei Wu will reflect on her over three-decade-long career as a gerontologist, exploring how her personal history, educational background, and professional experiences have informed and developed her program of research, focusing on her efforts to advance global healthy aging. Dr. Wu intends to highlight key findings from her research. She will discuss the pivotal role of secondary data analyses as a means to investigate the complex relationships between oral health, cognitive decline, and systemic health. Moreover, she will elaborate on the development of behavioral interventions aimed at improving oral health for individuals with cognitive impairment. She will also address her contributions to the establishment of NIH-funded centers aimed at mitigating health disparities and enhancing equity among older Asian Americans. Lastly, Dr. Wu will explore the policy and clinical ramifications derived from these findings, highlighting their potential to shape future approaches to gerontological care.

